# Potential Role of GST-*π* in Lung Cancer Stem Cell Cisplatin Resistance

**DOI:** 10.1155/2021/9142364

**Published:** 2021-11-19

**Authors:** Wenjun Wang, Jianping Wei, Xiaoyun Tu, Xiaoqun Ye

**Affiliations:** ^1^Department of Respiratory Diseases, The Second Affiliated Hospital of Nanchang University, Nanchang, Jiangxi 330006, China; ^2^Department of Respiratory Diseases, Ganzhou Affiliated Hospital of Nanchang University, Ganzhou, Jiangxi 341000, China

## Abstract

**Background:**

Cancer stem cells (CSCs) are responsible for tumorigenesis, chemoresistance, and metastasis. Chemoresistance is a major challenge in the management of lung cancer. Glutathione-sulphur-transferase-*π* (GST-*π*) plays an important role in the origin and development of various types of cancer by regulating the cellular redox balance. Recent investigations have demonstrated that GST-*π* is associated with the chemoresistance of lung CSCs (LCSCs). However, the mechanism of GST-*π* in lung cancer, particularly in LCSCs, remains unclear. The present study is aimed at exploring the potential role of GST-*π* in stemness and cisplatin (DDP) resistance of LCSCs. *Materials and methods*. In the present study, lung cancer cell spheres were established using the A549 cell line, which according to our previous research, was confirmed to exhibit characteristics of stem cells. Next, GST-*π* protein expression, apoptosis percentage, and intracellular reactive oxygen species (ROS) concentration in A549 adherent cells and A549 cell spheres were analyzed by western blotting and flow cytometry, respectively. Finally, DDP resistance, ROS concentration, and GST-*π* expression in LCSCs were analyzed following the interference with GST-*π* using DL-buthionine-(S,R)-sulphoximine and N-acetylcysteine.

**Results:**

The results revealed that GST-*π* was highly expressed in A549 cell spheres compared with A549 adherent cells and was associated with a decreased intracellular ROS concentration (both *P* < 0.05). Regulating GST-*π* protein expression could alter DDP resistance of LCSCs by influencing ROS.

**Conclusion:**

These results suggested that GST-*π* may be important for LCSC drug resistance by downregulating ROS levels. These findings may contribute to the development of new adjuvant therapeutic strategies for lung cancer.

## 1. Introduction

Lung cancer is the most frequent cause of tumor-related mortality worldwide. In particular, non-small-cell lung cancer (NSCLC) accounts for ~85% of all lung cancer cases [1]. The chemoresistance of tumor tissues limits the efficacy of chemotherapy and is currently a significant clinical problem. Recent studies have suggested that tumor chemoresistance involved numerous pathways and molecular mechanisms, including oncogenes (such as EGFR, PI3K/AKT, Erk, and NF-*κ*B), tumor suppressor genes (such as p53), drug transporter pumps, mitochondrial alterations, DNA damage and repair, autophagy, epithelial-mesenchymal transition, cancer stemness, and exosomes [2]. Take drug transporter pumps as example, chemotherapeutic drugs were pumped out by drug transport pumps actively pump form cancer cells, leading to the need for higher drug concentrations to achieve the same killing tumor effect. The “cancer stem cell (CSC) theory” posits that CSCs are a small population of cells in tumors, which have self-renewal and multidirectional differentiation potential [3, 4]. Of note, lung CSCs (LCSCs) play an important role in drug resistance in lung cancer and may serve as a target for cancer treatment [5, 6]. Our team has successfully isolated lung adenocarcinoma CSCs, which have unique energy metabolism characteristics, such as low mitochondrial DNA copy number, low intracellular reactive oxygen species (ROS) concentration, and low glucose and oxygen consumption [7, 8]. Previous studies have shown that the dynamic maintenance of ROS homeostasis, particularly ensuring low ROS concentrations, was required for stem cell protection, such as the induction of the breast CSC phenotype and preventing leukemic stem cell differentiation [9, 10] to protect stem cells. Researchers also found that maintaining a low intracellular ROS concentration in CSCs was closely associated with high drug resistance in previous studies [8]. Given these findings, CSCs were suggested to represent a robust model for investigating the relationship between chemotherapy resistance and ROS *in vitro*.

Glutathione (GSH) metabolism is known to play both beneficial and pathogenic roles in various malignancies. For example, it has been shown to help remove and detoxify carcinogens in cells; however, excess GSH promotes tumor progression and has been associated with increased metastasis [11]. GSH-sulphur-transferase (GST) is one of the enzyme antioxidants produced by the reaction catalysed by GSH, of which there are six different subtypes. However, this role of GST appears to be different in tumor and normal body metabolic processes [11]. GST-*π* functions as an antioxidant that participates in cell metabolism and can reduce ROS damage in cells. It has been demonstrated that the overexpression of the GST-*π* protein enhances chemoresistance in various types of cancer, including oesophageal squamous cell carcinoma [12], adenocarcinoma of the gastroesophageal junction [13], and squamous cervical, ovarian and lung cancer [14]. Our previous study revealed that GST-*π* was associated with the stemness of LCSCs [15]. A previous study also demonstrated that GST-*π* was associated with low concentrations of ROS in cancer cells [16]. In addition, Ding et al. [17] found that as GST-*π* expression decreased, ROS levels were elevated, cell viability was depressed, and apoptosis was increased in hepatocellular CSCs. Moreover, studies have reported multiple CSC markers that have been associated with chemoresistance in several first-line anticancer therapies, including aldehyde dehydrogenase 1, CD133, side population (Hoechst-negative), CD44, CD87, and CD117 [2, 6]. Based on the association between LCSCs and drug resistance and the important role of GST-*π* in LCSCs, it was hypothesized that GST-*π* may regulate the drug resistance of LCSCs through ROS.

The aforementioned studies demonstrated the important role of GST-*π* as a chemoresistance regulator and suggested the potential of GST-*π* in becoming a promising target for tumor treatment. Cisplatin (DDP) remains the first-line treatment option for various types of cancer; however, chemoresistance to this drug, which develops during cancer treatment, limits its effectiveness. To date, transporter pumps, long noncoding RNAs, microRNAs, the autophagy pathway, and metabolic reprogramming have been found to be involved in DDP resistance in NSCLC [18]. However, little information is available on the detailed mechanism between high GST-*π* expression in LCSCs and DDP resistance. In the present study, GST-*π* expression was analyzed in A549 LCSCs, and DL-buthionine-(S,R)-sulphoximine (BSO) and N-acetylcysteine (NAC), which are antioxidant and oxygen boosters, respectively, were used to regulate GST-*π* expression, as previously described [19, 20]. Next, intracellular ROS concentrations and cell apoptosis were detected to determine the potential resistance mechanism of NSCLCSCs to DDP. It was hypothesized that regulating GST-*π* and interfering with ROS concentration could influence DDP resistance in A549 tumor spheres. These results may provide a new prospective strategy for combining drugs that could avert or decrease drug resistance and avoiding those that increase drug resistance, in order to improve the efficacy of lung cancer chemotherapy.

## 2. Materials and Methods

### 2.1. Reagents and Antibodies

BSO (cat no. B2515), EGF (cat no. RP10914), and basic fibroblast growth factor (bFGF; cat no. RP8627) were obtained from Merck KGaA. Insulin (cat no. 9004108) was purchased from Jiangsu Wanbang Biochemical Pharmaceutical Co., Ltd. GST-*π* rabbit polyclonal antibody (cat no. A00394), whose epitope was a peptide corresponding to a sequence at the C-terminus of human GST-*π*, was obtained from Wuhan Boster Biological Technology, Ltd. Primary polyclonal antibodies against *β*-actin (cat no. AF5006), whose epitope was a peptide corresponding to a sequence at the N-terminus, and goat anti-mouse/rabbit monoclonal IgG (H&L; cat no. A0208) were purchased from Beijing TDY Biotech Co., Ltd. NAC (cat no. C84605) was obtained from Beijing Solarbio Science & Technology Co., Ltd. DDP (cat no. H20010743) was purchased from Jiangsu Haoshen Pharmaceutical Co., Ltd. The ROS Assay kit (cat no. S0033) and Annexin V/PI Apoptosis kit (cat no. 70APCC101100) were obtained from MultiSciences (Lianke) Biotech Co., Ltd. All reagents were commercially available and used according to the manufacturers' protocol.

### 2.2. Cell Culture and CSC Sphere Formation

The A549 cell line was obtained from the American Type Culture Collection and preserved at the Laboratory of the Molecular Centre of the Second Affiliated Hospital of Nanchang University (Nanchang, China). DMEM (cat no. SH3024301) and FBS (cat no. SH3008403) were purchased from Cytiva. B27 (cat no. 17504004) and DMEM/F12 (cat no. C11330500BT) were obtained from Gibco, Thermo Fisher Scientific, Inc. A549 cell spheres were cultured as described in our previous studies [7, 8]. Briefly, the A549 cell line was cultured in DMEM supplemented with 10% FBS and incubated at 37°C under a humidified atmosphere with 5% CO_2_. After 1~2 weeks, different single-cell-derived clones, which included holoclones and paraclones, had formed. To distinguish holoclones form all kinds of clones, fluorescence microscopy was used to detect the ROS fluorescence values of A549 single-cell colonies in different forms. Next, holoclones were isolated using cloning cylinders and cultured with serum-free medium (SFM) containing DMEM/F12, recombinant EGF, bFGF, insulin, and B27. A549 cells were seeded into 6-well plates for another 2 weeks at a density of 1 × 10^3^ cells per well to generate primary A549 cell spheres. The primary A549 cell spheres varied in size from dozens of cells to hundreds of cells were loosely structured and had an irregular shape [7]. The primary A549 cell spheres were trypsinized, and single cells were cultured for 1-2 weeks to form the secondary spheres. The secondary A549 cell spheres were collected for the following experiments.

### 2.3. Cell Treatments

Cells were plated into 12-well plates for 24 h and subjected to different treatments. In the first stage, A549 cells and A549 stem cell spheres were treated with different drugs. Cells were divided into four groups: (i) control A549 cells, (ii) DDP-treated A549 cells, (iii) control A549 stem cell spheres, and (iv) DDP-treated A549 stem cell sphere. All DDP groups received DDP at a final concentration of 0.042 mmol/l for 24 h. At the second stage, only A549 stem cell spheres were used. The (i) control group, (ii) DDP group, (iii) the BSO+DDP group, and (iv) the NAC+DDP group were created, according to different drug combinations. The DDP group was the same as previously described. The BSO+DDP group was treated with BSO at a final concentration of 1 mmol/l and DDP at a final concentration of 0.042 mmol/l for 24 h. The NAC+DDP group was treated with NAC at a final concentration of 5 mmol/l for 2 h, followed by a final concentration of 0.042 mmol/l DDP for 24 h.

### 2.4. Western Blotting

Total GST-*π* protein of A549 cells or A549 cell spheres was extracted by RIPA buffer, according to the manufacturer's instructions. After determining the protein concentration using a BCA protein assay kit, the protein was denatured and stored at -20°C. A mass of 40 *μ*g GST-*π* protein was subjected to 8% SDS-PAGE and transferred to a PVDF membrane. The membrane was blocked with 5% nonfat milk for 2 h and then incubated with primary antibodies (anti-GST-*π* rabbit polyclonal antibody diluted at 1 : 300 and anti-*β*-actin mouse polyclonal antibody diluted at 1 : 1,000) overnight at 4°C. Following washing thrice, the membranes were incubated with secondary antibodies at room temperature for 2 h. Finally, the protein bands were visualized with the ECL detection system, and the relative protein expression was normalized to *β*-actin expression using the ImageJ software (National Institutes of Health).

### 2.5. Flow Cytometry

Intracellular ROS concentration and apoptosis were both detected using flow cytometry. Cells were plated into 12-well plates for 24 h and exposed to different treatments. The cells were then resuspended in 3 ml diluted DCFH-DA (final concentration, 3.3 *μ*mol/l) and incubated with reagents of the ROS Assay kit, according to the manufacturer's instructions. Intracellular ROS concentrations were analyzed by flow cytometry. For apoptosis detection, the cells were collected from the culture medium and resuspended in PBS. The cells were then incubated with reagent from the Annexin V/PI apoptosis kit, according to the manufacturer's instructions, prior to flow cytometry.

### 2.6. Statistical Analysis

The results are expressed as the mean ± SD of three independent experiments. The statistical differences among different groups were analyzed using a one-way ANOVA with Bonferroni post hoc test. *P* < 0.05 was considered to indicate a statistically significant difference. Data quantification was performed using the ImageJ software, and figures were plotted in GraphPad Prism 5 (GraphPad Software, Inc.). Statistical analysis was performed using the SPSS 20.0 software (IBM Corp.).

## 3. Results

### 3.1. Generation of A549 Cell Spheres

**Figure 1:**
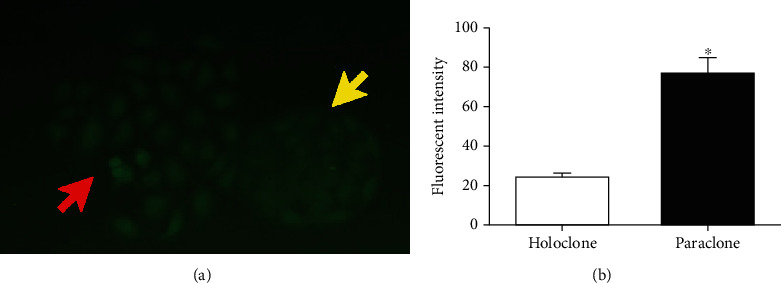
Screening for A549 stem cell spheres. (a) Two types of colonies were formed by the A549 cell line (magnification, ×400). The yellow arrowhead indicates a holoclone and the red arrowhead a paraclone. (b) Fluorescence mean values were used to detect the ROS concentration of A549 cell colonies in different forms. Data are presented as the mean ± SD of three independent experiments. *P* < 0.05 vs. the holoclone group. ROS: reactive oxygen species.

The A549 cell spheres were cultured with SFM and could form tumor spheres enriched for stem-like cells, as described in our previous studies [7, 8]. According to these previous experiments, mitochondrial and energy metabolism-related properties were used as indicators of LCSCs. Herein, we determined the intracellular ROS concentration and screening of the holoclones from different types of colonies, including holoclones and paraclones. After the ROS probe (DCFH-DA) was successfully loaded, the fluorescence value of the cells, which belonged to different clones, was detected. Clone fluorescence value was positively correlated with the concentration of ROS accumulated in the cells; thus, it indirectly reflects the concentration of ROS in the cells [8]. As shown in Figure 1(a), cells in holoclones exhibited a weaker fluorescence compared with paraclones under a fluorescence microscope. The fluorescence mean value of the holoclones (24.13 ± 1.79) was lower than that of the paraclones (77.89 ± 6.71; *P* < 0.05; Figure 1(b)), indicating that the holoclones had a lower ROS concentration, which suggested a higher number of CSCs than that of paraclones. This method of inducing and selecting stem cells has been repeatedly confirmed in our previous studies, and therefore, the confirmation of the stem cell phenotype was not repeated in this study.

### 3.2. Expression of GST-*π* Protein Is Associated with the Stemness of A549 Cell Spheres

**Figure 2:**
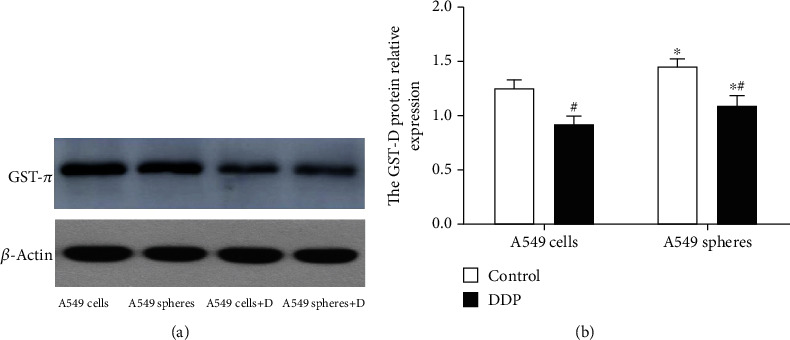
GST-*π* protein expression in A549 cells and A549 cell spheres. A549 cells and A549 cell spheres were treated with normal saline and DDP. (a) Protein expression of GST-*π* was detected by western blotting. (b) GST-*π* protein was normalized against *β*-actin. Data are presented as the mean ± SD of three independent experiments. ^∗^*P* < 0.05 vs. A549 cells; ^#^*P* < 0.05 vs. the control group. GST-*π*: glutathione-sulphur-transferase-*π*; DDP: cisplatin.

**Figure 3:**
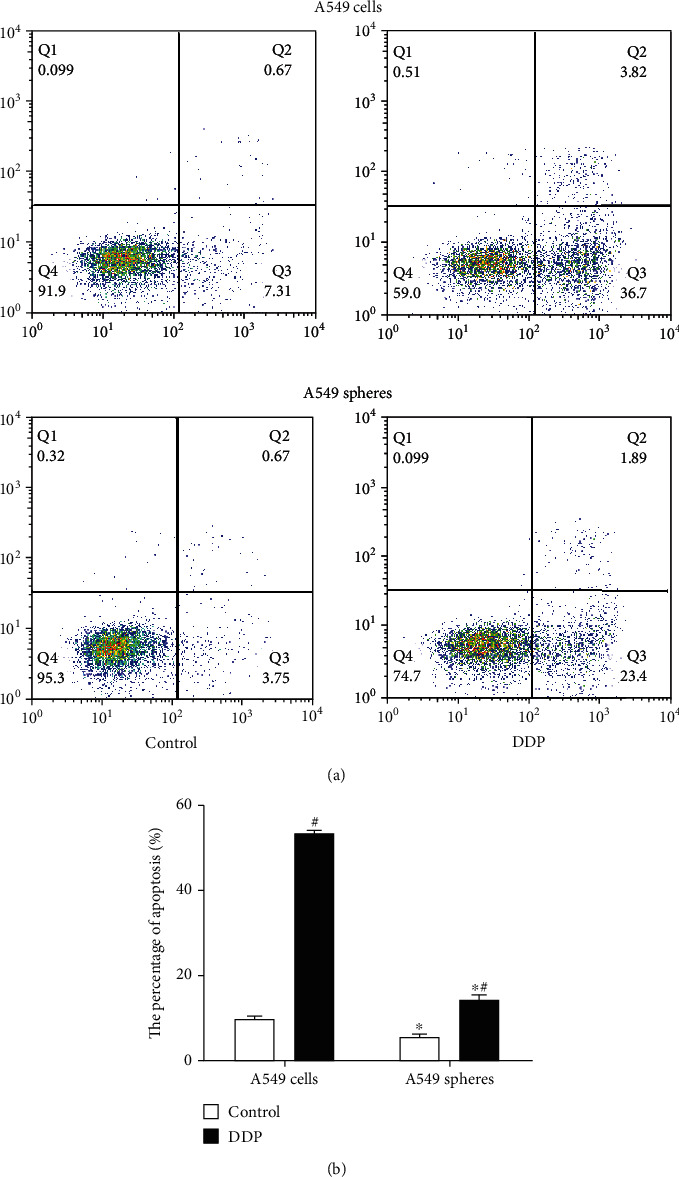
A549 cells and A549 cell spheres were treated with normal saline and DDP. The cells were divided into four groups: (i) control A549 cells, (ii) control A549 spheres, (iii) DDP-treated A549 cells, and (iv) DDP-treatment A549 cell spheres. (a) Cells were stained with an Annexin V/PI apoptosis kit, and the rate of apoptosis was analyzed by flow cytometry. (b) Percentage of A549 cells and A549 sphere cells undergoing apoptosis following different treatments. Data are presented as the mean ± SD of three independent experiments. ^∗^*P* < 0.05 vs. A549 cells; ^#^*P* < 0.05 vs. the control group. DPP: cisplatin.

**Figure 4:**
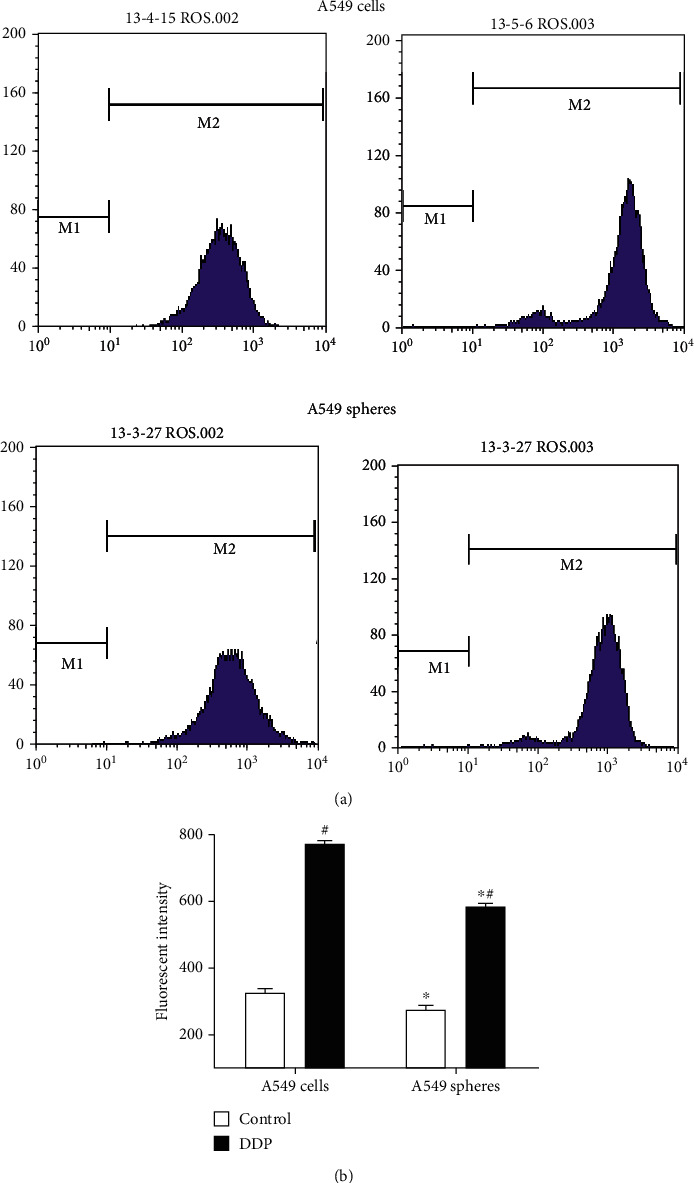
A549 cells and A549 cell spheres were treated with normal saline and DDP. The cells were divided into four groups: (i) control A549 cells, (ii) control A549 spheres, (iii) DDP-treated A549 cells, and (iv) DDP-treatment A549 cell spheres. (a) ROS levels of A549 cells and A549 cell spheres exposed to different treatments were detected by an ROS Assay kit, and the concentration was determined by flow cytometry. (b) ROS levels of A549 cells and A549 sphere cells with different treatments. Data are presented as the mean ± SD of three independent experiments. ^∗^*P* < 0.05 vs. A549 cells; ^#^*P* < 0.05 vs. the control group. DPP: cisplatin; ROS: reactive oxygen species.

As shown in our previous studies, A549 cell spheres displayed stemness properties [7, 8], such as lower ROS levels, differentiation potential, decreased levels of apoptosis, and increased chemotherapy resistance. According to our previous experiments and those of other studies focused on CSCs, GST-*π* was found to be associated with stemness properties [15, 17]. The GST-*π* protein expression was compared between A549 adherent cells and A549 cell spheres, and it was found that the protein expression in the A549 cell spheres (1.45 ± 0.07) was higher than in the A549 adherent cells (1.25 ± 0.06) (*P* < 0.05; Figure 2). And it was found that the DDP-treated A549 cell spheres (1.09 ± 0.09) had lower GST-*π* expression compared with the controls (1.45 ± 0.07), but a higher expression compared with the DDP-treated A549 adherent cells (0.91 ± 0.08; *P* < 0.05; Figure 2). To confirm the stemness of A549 cell spheres, the intracellular ROS concentration and levels apoptosis were also investigated. The percentage of apoptosis in the A549 cell spheres was clearly decreased compared with that of the A549 adherent cells (5.01 ± 0.31 vs. 9.79 ± 0.66%; *P* < 0.05; Figure 3). Similarly, the intracellular ROS concentration was lower compared with the A549 adherent cells (272.67 ± 16.04 vs. 326.33 ± 12.34; *P* < 0.05; Figure 4). Thus, whether GST-*π* was associated with lung cancer cell resistance to DDP was investigated. It was found that the percentage of apoptosis in A549 cell spheres treated with DDP (17.04 ± 1.55%) was slightly increased, as compared with that of the control group (9.79 ± 0.66%) but decreased compared with that of the DDP-treated A549 adherent cells (41.33 ± 1.28%; all *P* < 0.05; Figure 3). Furthermore, the ROS concentration of the DDP-treated A549 cell spheres (585 ± 12.12) was found to be clearly increased compared with the control group (272.67 ± 16.04) but decreased compared with the DDP-treated A549 adherent cells (768.67 ± 9.02) (all *P* < 0.05; Figure 3). These results showed that DDP-induced apoptosis in stem cells may be associated with ROS, and GST-*π*, as an antioxidant protein and a potential stem cell marker, may regulate ROS so as to DDP-induced apoptosis in stem cells.

### 3.3. GST-*π* May Regulate the Chemosensitivity of A549 Cell Spheres to DDP through ROS

**Figure 5:**
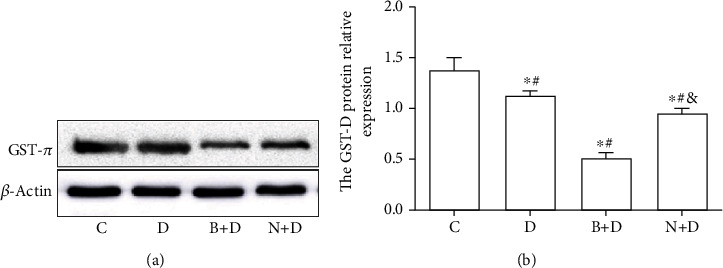
GST-*π* protein expression in A549 cell spheres. A549 cell spheres were treated with DDP, BSO combined with DDP, and NAC combined with DDP. (a) Protein expression of GST-*π* was detected by western blotting. (b) GST-*π* protein was normalized to *β*-actin. Data are presented as the mean ± SD of three independent experiments. ^∗^*P* < 0.05 vs. the control group; ^#^*P* < 0.05 vs. the D group; ^&^*P* < 0.05 vs. the B+D group. GST-*π*: glutathione-sulphur-transferase-*π*; DDP: cisplatin; BSO: DL-buthionine-(S,R)-sulphoximine; NAC: N-acetylcysteine.

**Figure 6:**
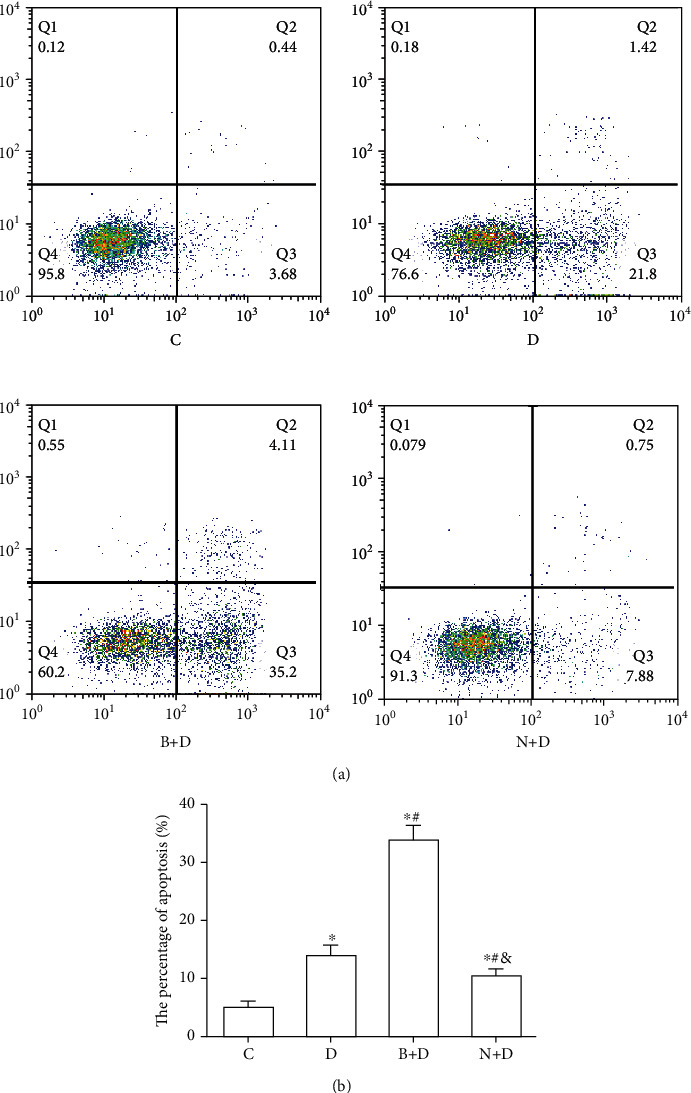
A549 cell spheres were treated with DDP, BSO combined with DDP, and NAC combined with DDP. (a) The apoptotic rate was analyzed by flow cytometry with an Annexin V/PI apoptosis kit among the C, D, B+D, and N+D groups. (b) Percentage of A549 sphere cells undergoing apoptosis upon different treatments. Data are presented as the mean ± SD of three independent experiments. ^∗^*P* < 0.05 vs. the control group; ^#^*P* < 0.05 vs. the D group; ^&^*P* < 0.05 vs. the B+D group. DDP: cisplatin; BSO, DL-buthionine-(S,R)-sulphoximine; NAC; N-acetylcysteine.

**Figure 7:**
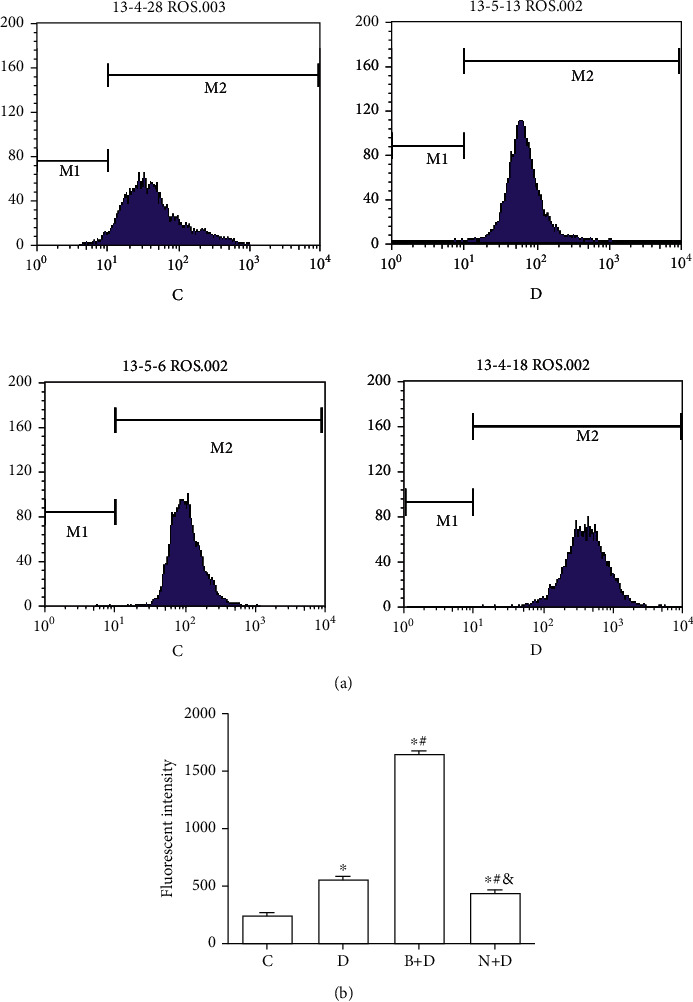
A549 cell spheres were treated with DDP, BSO combined with DDP, and NAC combined with DDP. (a) The ROS levels of A549 cell spheres exposed to different treatments were detected by an ROS Assay kit, and the concentration was determined by flow cytometry among the C, D, B+D, and N+D groups. (b) ROS levels in A549 sphere cells among different treatments. Data are presented as the mean ± SD of three independent experiments. ^∗^*P* < 0.05 vs. the control group; ^#^*P* < 0.05 vs. the D group; ^&^*P* < 0.05 vs. the B+D group. DDP: cisplatin; BSO: DL-buthionine-(S,R)-sulphoximine; NAC: N-acetylcysteine; ROS: reactive oxygen species.

To explore the association between GST-*π* and chemotherapy resistance in A549 stem cell spheres, the following groups were used: (i) control (C), (ii) DDP (D), (iii) BSO and DDP (B+D), and (iv) NAC and DDP (N+D) groups. The intracellular ROS concentration (251.65 ± 21.35) and apoptosis percentage (4.76 ± 0.84%) of control group were significantly lower than other groups, and the GST-*π* protein expression (1.38 ± 0.12) of control group was higher than other groups (all *P* < 0.05; Figures 5–7). Compared with the intracellular ROS concentration (585.00 ± 12.12) and apoptosis percentage (15.01 ± 1.08%) of the D group, the values in the B+D group were significantly increased (1,651.00 ± 28.62 and 32.11 ± 2.32%, respectively; all *P* < 0.05; Figures 6 and 7). However, the GST-*π* protein expression in the B+D group (0.51 ± 0.05) was clearly decreased compared with the D group (1.12 ± 0.05; *P* < 0.05; Figure 5). Compared with those in the D group, the ROS levels (452.33 ± 16.16) and apoptosis percentage (9.97 ± 1.74%) in the N+D group were decreased (all *P* < 0.05; Figures 6 and 7). Of note, the GST-*π* protein expression (0.95 ± 0.04) was still slightly decreased in the N+D group (*P* < 0.05; Figure 5), which was inconsistent with the expected results. In addition, the GST-*π* protein expression in the N+D group was significantly higher compared with the B+D group (*P* < 0.05; Figure 5), and the ROS concentration and apoptosis percentage were significantly decreased in the N+D group compared with the B+D group (all *P* < 0.05; Figures 6 and 7). All these outcomes indicated that GST-*π* may regulate the concentration of intracellular ROS, which in turn affect the efficacy of DDP in LCSCs. As an important and frequently used drug in lung cancer, NAC may affect the proapoptotic effect of DDP through GST-*π* and this may occur, not only through by affecting ROS, but also by affecting other signalling pathways.

## 4. Discussion

As a result of the in-depth research on CSCs, a consensus has been reached on the CSC hypothesis [4–6, 21]; that is, CSCs may be the cause of cancer resistance, recurrence, or distant metastasis and could therefore serve as a target in clinical management. In the serum-free medium, in the process of inducing A549 stem cells, holoclones were selected as it was considered that holoclones contained more stem cells. Differences in ROS concentration within the cell reflect differences in the content of stem cells in different types of clones [8]. Determining the ROS concentration further confirmed the feasibility of this method to isolate stem cells. Stem cell phenotype identification was repeatedly confirmed in our previous studies and was therefore not repeated herein. In the present study, it was found that, compared with adherent A549 adherent cells, A549 cell spheres presented an enhanced resistance to DDP treatment, a lower intracellular ROS concentration, and decreased apoptosis, findings consistent with those of our previous study [8]. These results suggested that A549 cell spheres are robust tools for investigating chemoresistance in NSCLC. In addition, based on previous studies which found GST-*π* was significantly enhanced in sphere-derived cells, the present study focused on the role of GST-*π* in stem cells. In the present study, it was demonstrated that GST-*π* may be associated with the stemness and drug resistance of A549 tumor spheres, and ROS generation may be the main underlying mechanism. Regulating the expression of GST-*π* may lead to the regulation of the sensitivity of A549 spheres to DDP.

GST-*π* is an important protein of the enzyme antioxidant defence systems participating in cell proliferation and apoptosis by regulating redox [16]. An elevated expression of GST-*π* is often observed in solid tumor tissues, with GST-*π* considered as an oncogene essential for the occurrence, metastasis, and drug resistance of cancer [12, 13, 22–24]. Dong et al. [25] demonstrated that high GST-*π* expression maintained the resistance of breast cancer cells to adriamycin by promoting autophagy. A study of 104 patients with colorectal cancer receiving fluoropyrimidine and platinum-based chemotherapy regimens suggested that the genetic polymorphisms of GST-*π* were closely associated with chemotherapy effects and adverse reactions, suggesting that GST-*π* plays a role in the mechanism of action of different types of chemotherapy drugs [26]. Other studies have suggested that a high expression of GST-*π* was closely associated with certain carcinogenic characteristics of CSCs [27], such as increased cell viability [17], low intracellular ROS [16, 17], and DDP resistance [15, 28], which can lead to the development of chemoresistance and tumor relapse. It was also found that the antitumor effect of DDP may depend on the increased levels of intracellular ROS. In addition, it was observed that GST-*π* protein expression in A549 stem cell spheres was higher than that in A549 adherent cells and maintained the DDP-induced low intracellular ROS concentrations and reduced apoptosis levels. In combination, these results suggested that GST-*π* is at least partly involved in maintaining the stemness of LCSCs.

The degree of activation and increase in ROS appears to correlate with the cell line's sensitivity to DDP [29]. However, only a few studies have been conducted on the resistance of ROS to DDP in LCSCs, with the specific mechanism still unclear. Follow-up research focused on whether GST-*π* regulates the resistance of stem cells to DDP through the regulation of ROS is required. Previous studies have shown that the downregulation of GST-*π* is associated with the increased sensitivity of lung cancer cells to DDP [14, 15]. BSO, an oxygen promoter, can specifically inhibit the activity of *γ*-glutamate cysteine synthase, the key rate-limiting enzyme in GSH synthesis, and downregulate the expression of GSH/GST-*π*. In breast CSCs, the scavenging ability of intracellular ROS decreased following BSO pretreatment, which may contribute to tumor radioresistance [30]. BSO altered ROS levels in A549 lung cancer cells following carboplatin-induced cell killing, while NAC had the opposite effect [20]. As an antioxidant, NAC is deacetylated after entering the cell to form L-cysteine, which helps protect cells from cytotoxic damage caused by low levels of GSH [31]. Stewart et al. [32] confirmed that DDP significantly increased intracellular ROS and promoted apoptosis in malignant pleural mesothelioma cells, an effect that could be reversed by NAC. A study demonstrated that, in both mouse and human lung cancer cells, NAC enhanced cancer cell proliferation by reducing ROS, aggravating DNA damage, and increasing p53 expression [19]. In high-risk populations, such as smokers and patients with chronic obstructive pulmonary disease receiving NAC to alleviate mucus generation, the antioxidant NAC may accelerate the formation of early cancer or the development of precancerous lesions [19]. However, few studies have investigated whether NAC upregulates GST-*π* to increase ROS and eliminate DDP, leading to the apoptosis of LCSCs. The present results suggested that BSO inhibited chemoresistance in A549 cell spheres through the downregulation of GST-*π* and increased ROS levels, while NAC had the opposite effect. Therefore, evidence was provided herein that GST-*π* regulated the DDP resistance of LCSCs by transforming the intracellular redox balance, namely, ROS. Nevertheless, although NAC decreased the concentration of ROS and increased the chemoresistance of A549 cell spheres, it was not completely consistent with the protein expression levels of GST-*π*, indicating that NAC might affect ROS and apoptosis through other mechanisms. As a result of the dual nature of NAC that antagonizes the activity of proteasome inhibitors and inhibits ROS, a previous study demonstrated that data interpretation may not be straightforward when NAC is used as an antioxidant to demonstrate ROS involvement in drug-induced apoptosis [33]. The study just described perhaps helped to interpret our last outcome that GST-*π* was inconsistent with ROS generation and apoptosis percentage; however, further studies are required.

The present study was not without its limitations. For example, adherent cells and stem cell pellets were only treated with one drug concentration. Although previous studies have provided satisfactory results, further research is required to explore whether DDP management to cells is equal to every single cell. In combination, the results of the present study exhibited an increased chemoresistance in LCSCs, which was associated with a high protein expression of GST-*π* and low intracellular ROS concentrations. It was also shown herein that regulating the expression of GST-*π*, thereby increasing the concentration of ROS, could resensitize LCSCs to chemotherapy. Consequently, this study may have provided a promising treatment for patients with NSCLC.

## Data Availability

The data used to support the findings of this study are available from the corresponding author upon request.
